# Detection of p53 mutations in precancerous gastric tissue

**DOI:** 10.1038/sj.bjc.6601302

**Published:** 2003-09-30

**Authors:** C Morgan, G J S Jenkins, T Ashton, A P Griffiths, J N Baxter, E M Parry, J M Parry

**Affiliations:** 1 Human Molecular Pathology Group, Swansea Clinical School, University of Wales Swansea, Singleton Park, Swansea SA2 8PP, UK; 2 Department of Health and Exercise Science, De Montfort University, Landsdowne Road, Bedford MK40 2BZ, UK; 3 Pathology Department, Morriston Hospital, Morriston, Swansea, UK; 4 School of Biological Sciences, University of Wales Swansea, Singleton Park, Swansea SA2 8PP, UK

**Keywords:** p53, gastric cancer, gastritis, intestinal metaplasia, reactive oxygen species, *Helicobacter pylori*

## Abstract

Intestinal-type gastric cancer is preceded by gastritis and intestinal metaplasia. There is uncertainty regarding the stage at which genetic alterations in the p53 gene occur. Reactive oxygen species (ROS) may participate in the production of mutations and the inactivation of p53 is due to infection by the bacterium *Helicobacter pylori.* We have investigated whether alterations of the p53 gene can be detected in gastritis and intestinal metaplasia using the restriction site mutation assay. We also assessed the potential contribution of ROS to p53 inactivation using electron spin resonance spectroscopy (ESR) and correlated with the presence of *H. pylori*. In all, 35% of the gastritis samples and 45% of the intestinal metaplasia samples were found to contain mutations in exons 5–8 of the p53 gene. Electron spin resonance spectroscopy analysis showed a significant increase in free radical levels in gastritis samples compared with normal, intestinal metaplasia and cancer samples, suggesting that free radicals present in gastritis may contribute to p53 mutations. There was no significant difference in free radical levels between the *H. pylori*-positive and -negative groups. However, a small subpopulation of the *H. pylori*-negative patients had much higher levels of free radicals. This suggests a more prominent role for other factors in ROS production.

The p53 gene is the most frequently inactivated tumour suppressor gene identified in human cancers to date (Han *et al*, 2002). Over 15 000 p53 mutations have been documented from tumour and cell line samples (www.iarc.fr), with loss of p53 function most commonly induced through point mutation (Bates and Vousden, 1996). More than 30% of p53 mutations occur at methylated CpG sites in codons 157, 175, 245, 248, 273 and 282 (Chen *et al*, 1998). Thus, CpG sites frequently act as mutational hot spots, which warrant their investigation to determine their potential role in tumour aetiology. p53 gene mutations appear to be key factors in the development of gastric cancer (Gomyo *et al*, 1996) having been documented in more than 60% of the reported cancer cases (Tamura *et al*, 1991). Classically, gastric cancer can be divided into two histological subtypes: diffuse or intestinal (Lauren, 1965). The intestinal type is believed to arise from a sequence of gastritis, intestinal metaplasia and increasing grades of dysplasia. There is controversy regarding the stage at which genetic alterations of the p53 gene occur within the metaplasia – dysplasia sequence (Uchino *et al*, 1993; Becker *et al*, 2000). p53 mutations detected in gastritis have been documented by Stemmermann *et al* (1994) and Kodama *et al* (1998), while studies by Zheng and YouYoung (1998), Shiao *et al* (1994) and Ochiai *et al* (1996) show p53 alterations only in intestinal metaplasia. Mutations of p53 in early gastric cancer have been found by Tohodo *et al* (1993) and Uchino *et al* (1993), while Romitti *et al* (1998), Brito *et al* (1994) and Joypaul *et al* (1993) claim that p53 mutations are late events in gastric cancer. However, p53 alterations in these studies have been detected using a variety of methods such as the polymerase chain reaction (PCR), PCR – single-strand conformation polymorphism, direct sequencing and immunohistochemistry. Thus, it appears that the frequency and stage at which p53 alterations are detected may depend on the methods used to detect them (Stemmermann *et al*, 1994). To investigate the timing of p53 mutations in gastric cancer, we employed the restriction site mutation (RSM) assay (Myers and Parry, 1994). This method detects mutations at restriction enzyme sites in human genes. Fortuitously, five of the eight main p53 mutational hot spots (codons 175, 213, 248, 249 and 282) contain restriction sites and are amenable to RSM analysis in patients with gastritis and intestinal metaplasia. Exhaustive digestion of the target DNA followed by amplification of the enzyme-resistant (mutated) sequences by PCR means that RSM is capable of detecting low levels of mutations (one mutated sequence among 10^4^ – 10^5^ wild-type sequences) (Jenkins *et al*, 1999). This, in turn, allows for the molecular selection of mutated samples and makes mutation detection in premalignant samples feasible.

As the bacterium *Helicobacter pylori* (*H. pylori)* has been implicated in gastric cancer progression through its induction of inflammatory-mediated reactive oxygen species (ROS), we assessed the *H. pylori* status of the patients using histology and PCR-based analysis. In addition, the level of ROS present in tissues representative of the different stages of the metaplasia – dysplasia sequence was directly measured in a separate cohort of gastric biopsies using electron spin resonance (ESR) spectroscopy. Electron spin resonance spectroscopy is currently the most sensitive, specific and direct method of measuring free radicals in tissue and body fluids (Ashton *et al*, 1999; Berliner *et al*, 2001).

## METHODS

### Materials for ESR

Antral biopsy samples were taken from consenting patients attending endoscopy. The ethical approval for this study was granted by the Local Research Ethics Committee. Biopsies were removed with standard gastric biopsy forceps and then cut in half with sterile scalpel blades. Half the biopsy sample was sent for histological examination, while the other was placed on ice for transportation back to the laboratory, and then stored at −20°C. Frozen samples were also obtained from fresh gastrectomy specimens. In total, 21 normal, 23 gastritis, 12 intestinal metaplasia and 13 carcinoma samples were analysed.

### Methods for ESR

A 140 mmol l^−1^ solution of the spin trap *α*-phenyl-tert butyl nitrone (PBN) (Sigma-Aldrich, Dorset, UK) was freshly prepared in sterile sodium chloride, and aliquoted into individual McCartney bottles. To prevent photolytic degradation and the generation of artefactual radicals, the PBN solution was kept in the dark and placed on ice until use. Following collection of samples at endoscopy, samples were weighed and then immediately incubated in the spin trap agent PBN for approximately 2 h at 37°C in a water bath. During this incubation, usually short-lived free radicals present in the biopsy samples were collected and was stabilised by the PBN solution. Organic extraction was carried out by adding the PBN solution to an equal volume of HPLC grade toluene (Sigma-Aldrich, Dorset, UK), which had been previously degassed for 2 h. The toluene/PBN solution was vortexed for 3 min and then centrifuged for 3 min at 13 000 rpm to separate the organic layer. The organic layer was then pipetted off into a new sterile McCartney bottle (covered in foil), ready for analysis. The organic layer containing the PBN adduct was transferred to a precision-bore quartz ESR sample tube which was then vacuum degassed in a freeze – pump – thaw procedure, using a turbo pump to 10^−3^ Torr for three consecutive 5 min cycles. The sample was then immediately analysed at room temperature using a Bruker EMX series X-band spectrometer at the University of Glamorgan, Pontypridd, UK.

### Materials for RSM

Biopsies for RSM were collected from a different cohort of patients because the toluene used in ESR analysis degrades DNA rendering it unsuitable for RSM analysis. Furthermore, whole biopsy samples were needed to provide sufficient DNA for RSM analysis. Thus, in the RSM study, paired biopsy samples were taken for DNA extraction and histology. Paraffin-embedded archival material was also used, which was obtained from the Histopathology Department of Morriston Hospital, Morriston, Swansea. In total, 12 normal samples (two fresh, 10 archival), 20 gastritis samples (eight fresh, 12 archival) and 20 intestinal metaplasia samples (six fresh, 14 archival) were analysed.

### DNA extraction for RSM

DNA extraction from biopsy samples was carried out using a high salt method (Stratagene, Cambridge, UK), while extraction of DNA from archival material was carried out using the DNeasy tissue kit (Qiagen, Crawley, UK).

### RSM analysis

In total, 1 *μ*g of DNA, containing 3 × 10^5^ copies of the p53 gene, from gastritis, intestinal metaplasia and control samples (diluted in 15 *μ*l of distilled H_2_O) was incubated with 2 *μ*l of the restriction enzyme of interest, 2 *μ*l *Taq* polymerase thermo buffer (Promega Corp., Southampton UK) supplemented with 1.25 mM MgCl_2_, overnight at the temperature optimum for enzymatic digestion. Polymerase chain reaction was carried out on the digested DNA samples by adding 15 pmol of each designated primer, 2.5 U of *Taq* polymerase (Promega Corp.), 1 × thermo buffer (Promega Corp.), 100 *μ*M of each dNTP (Promega Corp) and 1.5 mM MgCl_2_ to each tube, and the volume made to 50 *μ*l with H_2_O. Polymerase chain reaction primers and restriction enzymes used were: forward, 5′CCGCGCCATGGCCATCT; reverse, 5′GCGCTCATGGTGGGGG (*Hha*1) for exon 5, hot spot codon 175; forward, 5′GTCCCCAGGCCTCTGATTCCTC; reverse, 5′TAACCCCTCCTCCCAGAGACCCCAG (*Taq*1) for exon 6, hot spot codon 213; forward, 5′ATGTGTAACAGTTCCTGCATGG; reverse, 5′CTGACCTGGAGTCTTCCAGTG (*Msp*1/*Hae*III) for exon 7 hot spots 248/249 and forward, 5′CCTCTTGCTTCTCTTTTCCTATCC; reverse, 5′CTTGGTCTCCTCCACCGCTTCTTG (*Msp*1) for exon 8, hot spot codon 282.

The thermal cycle consisted of a preincubation step of 2 min at 94°C for complete denaturation of the DNA followed by 31 cycles (27 cycles for exon 7) at 94°C for 30 s, a specific annealing temperature of 60°C (exons 5, 7 and 8) or 65°C (exon 6) for 10 s and an extension phase at 72°C for 10 s. After PCR amplification, 16 *μ*l of PCR product was subjected to a second round of digestion with 2 *μ*l of the appropriate restriction enzyme and 2 *μ*l of enzyme-specific buffer overnight at the recommended temperature to remove any remaining wild-type DNA that may have escaped the initial digestion. Restriction site mutation products were then electrophoresed on 6 or 10% polyacrylamide gels (depending on the size of the PCR product) using a Protean III electrophoresis system (Biorad, Hemel Hempstead, UK) and stained with silver. Putative enzyme-resistant samples were detected as undigested bands corresponding to the same size as a positive (undigested) PCR control.

### Sequencing

Mutations detected by their resistance to enzyme digestion were confirmed by sequencing. Resistant PCR products of exon 5 (71 bp) and exon 7 (79 bp), which were too small for direct sequencing, were cloned into pCR2.1 plasmid vectors using a TA cloning kit (Invitrogen Corp., The Netherlands) prior to sequencing. Enzyme-resistant PCR products greater than 100 bp were sent for sequencing (Oswel, University of Southampton, UK). Only mutations evident on both strands of the DNA were deemed as clear positive results.

### *H. pylori* detection

Detection of *H. pylori* infection for the samples used in the ESR and RSM analysis was determined by histological examination by the same pathologist to maintain consistency. In addition, *H. pylori* infection in the gastritis samples, from the RSM study, was confirmed by PCR using forward primer 5′AAACCAATCGCTGTGAAACC and reverse primer sequences 5′ACGGAAGGCTTTCTCTCACA to generate a 94 bp fragment of the flagellin gene. The thermal cycle consisted of a preincubation step of 2 min at 94°C, 30 cycles at 94°C for 30 s, a specific annealing temperature of 60°C for 10 s and an extension phase at 72°C for 20 s. Gastritis samples identified as *H. pylori* positive were then subjected to further PCR analysis for detection of the cag A gene. Primers for the amplification of the cag A gene were synthesised according to Lage *et al* (1995) and the thermal cycle was the same as above, differing only in cycle number (35 cycles) and extension time (30 s).

### Statistical analysis

Data generated from the ESR samples were analysed using the nonparametric Mann – Whitney *U*-test for two group comparisons and the Kruskal – Wallis test and the Bonferroni procedures were carried out for multiple group comparisons. Results were considered statistically significant when *P*<0.05.

## RESULTS

### Electron spin resonance spectroscopy results

Table 1

**Table 1:** Mean number of radicals according to tissue type

**Tissue type**	**No. of samples**	**Mean no. of radicals (10^3^)**	**s.d.**	**s.e.**
Normal	21	6.102	4.737	1.033
Gastritis	23	8.456	8.532	1.779
Intestinal metaplasia	12	5.000	3.184	0.919
Cancer	13	3.361	1.489	0.413

shows the number of free radicals detected in the normal, gastritis, intestinal metaplasia and tumour samples. The Kruskal – Wallis test revealed a overall significant difference in the free radical levels (*P*=0.050) between all the groups. Two-group comparisons revealed a significant difference in free radical levels between gastritis and tumour samples and between normal and tumour sample. However, after applying the Bonferroni procedure the adjusted *P*-value required for significance was 0.008. This meant a significant difference was found in free radical levels only between gastritis and tumour samples, although an overall trend towards increased free radical levels in the gastritis samples was observed (Figure 1

**Figure 1:**
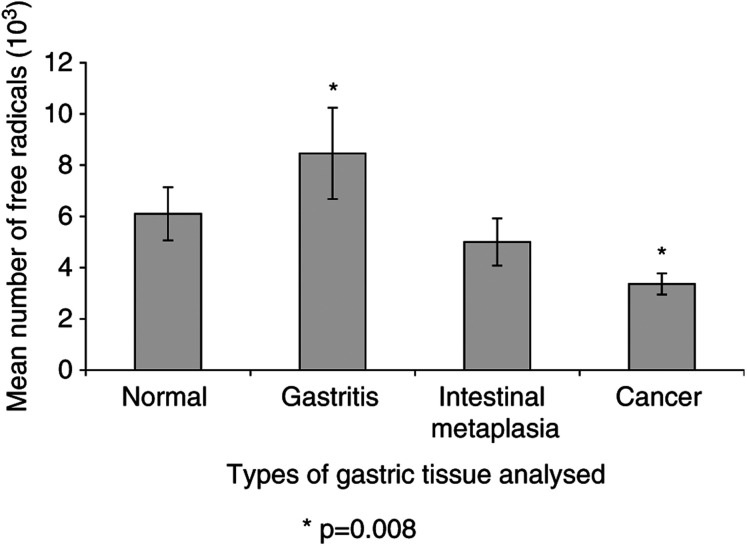
Mean number of radicals in precancerous and cancerous gastric tissue detected by ESR spectroscopy.

).

When the gastritis samples were subdivided into *H. pylori*-positive and -negative groups, using histology, ESR analysis revealed the levels of free radicals in the *H. pylori*-positive samples to be at a similar level to those in the negative group (Table 2

**Table 2:** Mean number of radicals with regards to *H. pylori* status

**Tissue type**	**No. of samples**	**Mean no. of radicals (10^3^)**	**s.d.**	**s.e.**
*H*. *pylori* positive	8	5.350	1.567	0.554
				
*H*. *pylori* negative	15	10.113	10.238	2.642

and Figure 2

**Figure 2:**
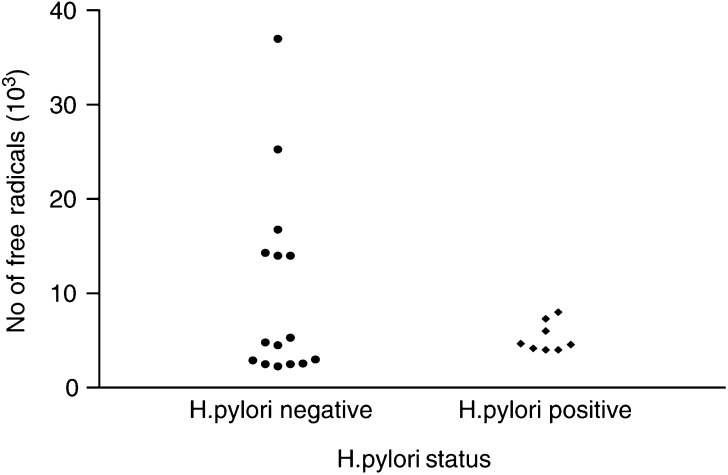
Free radical levels (10^3^) in *H. pylori*-positive and -negative gastritis tissue.

). Statistical analysis confirmed there was no significant difference in the levels of free radicals between the two groups (*P*=0.948). Interestingly, a trend towards increased free radical level was observed in the *H. pylori*-negative samples. Electron spin resonance spectroscopy analysis highlighted a subpopulation of these patients as having much higher levels of free radicals than those of the rest of the group or when in comparison to the *H. pylori*-positive sample data. Furthermore, gastritis samples were subdivided, again by histological examination, according to their degree of acute and chronic inflammation (Table 3

**Table 3:** Degree of inflammation in gastritis samples with regard to free radical level

**Patient no.**	**Acute inflammation**	**Chronic inflammation**	**No. of free radicals (10^3^)**
3	0	1	2.9
6	2	3	6
10	1	3	7.3
12	1	2	4.2
15	0	2	8
20	2	1	4.6
22	0	1	25.3
27	0	1	16.8
35	0	1	4.5
39	0	1	2.3
40	0	1	37
41	0	1	14
47	0	1	5.3
52	2	3	4.7
55	0	1	2.5
58	0	1	14
59	0	1	3
60	0	1	2.5
61	0	1	2.6
63	0	1	4
64	0	1	4
67	3	2	14.3
69	0	1	4.8

Degree of inflammation: 0=none, 1=mild, 2=moderate and 3=severe.

). A linear multiple regression analysis was carried out on this data to determine the extent to which acute or chronic inflammation (and their respective severity) could explain statistically the variation in free radical levels. No significant effect (*P*=0.972) was found with all variables in the equation. In addition, the *R*^2^ value of 0.096 suggests that only a small proportion, if any, of the variation in free radical levels could be explained by different grades of inflammation.

### Restriction site mutation results

No RSMs were detected in any of the p53 hot spot codons studied from the 12 normal samples. Thus, mutations found in subsequent samples were deemed to be genuine and not a result of experimental error. Of the 20 gastritis samples analysed, 35% (7 out of 20) of the samples were resistant to restriction enzyme digestion in one of the five codons studied, but eight mutations in total were found upon sequencing (with one sample having a double mutation in exon 7 at codons 249 and 250). Of the 20 intestinal metaplasia samples analysed, 45% (9 out of 20) of the samples were found to be resistant to restriction enzyme digestion, showing 11 mutations in total, with two samples containing a double mutation (one sample had mutations at codons 248 and 250 of exon 7 and the other sample contained a double mutation at codon 175 of exon 5 and codon 250 of exon 7) (Table 4

**Table 4:** p53 mutations detected in gastritis and intestinal metaplasia (Im) samples using RSM

**Exon**	**Codon**	**Mutation**	**Amino-acid change**	**Histology**
5	174	G → A	Silent	Gastritis
6	213	A → G	Silent	Gastritis
7	248	G → A	Arg → Gln	Gastritis
7	248	G → A	Arg → Gln	Gastritis
7	249	A → G	Arg → Gly	Gastritis
7	249	G → A	Arg → Lys	Gastritis
7	249	G → A	Arg → Lys	Gastritis
7	250	C → T	Pro → Ser	Gastritis
5	175	C → T	Arg → Cys	Im
7	247	C → T	Silent	Im
7	248	G → T	Arg → Leu	Im
7	248	G → A	Arg → Gln	Im
7	248	G → A	Arg → Gln	Im
7	249	A → G	Arg → Gly	Im
7	249	G → A	Silent	Im
7	250	C → T	Pro → Ser	Im
7	250	C → T	Pro → Ser	Im
7	250	C → T	Pro → Ser	Im
7	250	C → T	Pro → Ser	Im

). The majority of the mutations were clustered at hot spot codons 248, 249 and 250 of exon 7 (five mutations at each codon).

### Types of mutations detected by RSM

Of the 19 mutations detected in total, 15 were missense mutations resulting in an amino-acid change. Six of these missense mutations were detected in the gastritis samples and the remaining nine were detected in intestinal metaplasia samples. Of these missense mutations, one was a GC → TA transversion, two were AT → GC transitions and 12 were GC → AT transitions, with six occurring at CpG dinucleotides. The remaining four mutations were silent, producing no amino-acid change (Table 4).

### Detection of *H. pylori* infection

As *H. pylori* has been implicated in the gastritis stage of gastric cancer progression, biopsies from patients with gastritis (within the RSM study) were analysed by PCR to determine the subtype of *H. pylori* present (specifically, the cag A virulence factor).

Table 5

**Table 5:** *H. pylori*, cag A and p53 mutational status of gastritis samples

**Patient no.**	***H. pylori +/−***	**cag A +/−**	**Mutation**	**Codon**
5	+	+	GC → AT	249/250
7	+	−		
8	+	−		
9	+	−		
12	−	−	AT → GC	249
13	+	−		
24	+	+	GC → AT	248
25	+	+		
28	+	−	GC → AT	174
30	+	−	GC → AT	249
34	+	−		
71	+	+	AT → GC	213
72	+	−		
73	+	+		
74	+	+	GC → AT	248
75	+	+		
76	+	−		
77	−	−		
78	−	−		
79	−	−		

shows the gastritis samples and their corresponding *H. pylori*, cag A and p53 mutational status. Polymerase chain reaction analysis revealed that 80% (16 out of 20) of these samples were positive for *H. pylori*, which supports the current literature. From the 16 samples shown to be positive for *H. pylori* infection, 44% (7/16) were found to possess the cag A gene. Interestingly, six out of the seven individuals found to have p53 mutations were *H. pylori* positive. This suggests that *H. pylori* may be capable of causing p53 mutations in a subpopulation of individuals. Furthermore, the fact that four out of the six samples positive for *H. pylori* infection and p53 mutations also had the cag A gene would support the hypothesis that strains possessing cag A are more virulent. However, a contingency *χ*^2^ test showed no significant association between p53 mutations, *H. pylori* status and cag A status was observed (*P*=0.439), presumably as a consequence of the low numbers involved.

## DISCUSSION

The identification of individuals at a high risk of progressing to cancer offers promising possibilities for the prevention of cancer (Hussain and Harris, 1996), and in most cases the outcome largely depends on an early diagnosis (Matturri *et al*, 1998). However, little is still known about the genetic events responsible for the initiation and progression of gastric cancer (Koo *et al*, 2000; Sud *et al*, 2001). We have used the RSM assay and the ESR technique to analyse gastritis and intestinal metaplasia samples to address the issue of whether genetic alteration of p53 is an early or late event in gastric carcinogenesis, and to obtain a direct measure of free radical levels (on separate tissue samples) at each stage in the sequence of events leading to gastric cancer. As the *H. pylori* status was also known for these patients, we have also been able to correlate the *H. pylori* status with both the free radical load and the presence of a p53 mutation. The hypothesis we have been testing here is that *H. pylori*, and in particular those strains that possess the cag A virulence gene, induces ROS in gastric tissue which subsequently introduces genetic alterations (p53 mutations), which drive gastric cancer progression.

The RSM assay has previously been shown to have a detection limit of one mutated sequence in 10^−4^ – 10^−5^ wild-type sequences (Jenkins *et al*, 1999, 2001). Hence, this methodology is well suited to study premalignant tissue for the presence of early p53 mutations. p53 mutations were found in 0% of controls, 35% of gastritis samples and 45% of intestinal metaplasia samples. Of the 19 mutations detected in total, 63% (12 out of 19) were located at codons regarded as mutational hot spots by the IARC database (codons 175, 213, 248 and 249) and 15 were missense mutations resulting in amino-acid substitutions. The detection of p53 gene mutations in gastritis and intestinal metaplasia indicates that genetic alterations of p53 can be an early event in the pathogenesis of gastric cancer, and suggests that this approach may be suitable for detecting them. The types of p53 mutations (mainly GC → AT transitions) further suggest a role of ROS, which are known to favour such mutation types (Moraes *et al*, 1990; Feig *et al*, 1994; Purmal *et al*, 1994; Jenkins *et al*, 2001).

In an attempt to correlate the stage at which p53 mutations occur with the stage of cancer progression, ESR spectroscopy was used. In this separate study, there was a significantly increased level of free radicals in gastritis samples compared with normal, intestinal metaplasia and cancer. This is consistent with the hypothesis that ROS generated in inflamed tissue may be important in carcinogenesis. It is known that polymorphonuclear leucocytes can produce ROS during host defence reactions such as that associated with the bacterium *H. pylori.* When the gastritis samples were subdivided into *H. pylori*-positive and -negative samples, and their free radical levels compared, no significant difference in free radical levels was found. Furthermore, there was also no correlation between degree of inflammation and free radical levels. Interestingly, however, a small population of the samples within the *H. pylori*-negative group was shown to exhibit much higher levels than the rest of the group. This suggests a potential role for factors other than *H. pylori*, or even severity of gastric inflammation, to be the primary cause of ROS generation in gastric tissue and highlights this subpopulation as an interesting group in which to carry out further investigations.

The gastritis samples from the p53 study were also analysed to determine whether there was any relationship with *H. pylori* infection and the cag A virulence factor. No significant difference was shown to exist between samples in terms of their *H. pylori* and p53 mutational status. Nevertheless, p53 mutations were found in seven individuals, six of whom were positive for *H. pylori* infection, with four out of the six being cag A positive. This suggests that *H. pylori* infection (and subtype) may play a role in inducing mutations in the p53 gene. Therefore, this result may provide evidence for a causal relationship between ROS-induced mutations of p53 in inflamed tissue as a result of *H. pylori* infection, but only in certain individuals.

Here we have shown, using two different cohorts of patients, that p53 mutations are detectable in gastritis and that this stage also shows the highest levels of ROS. Our findings have also shown that although no significant difference in free radical levels were observed between *H. pylori*-infected and noninfected samples, the bacterium may contribute to genomic instability (shown here by the presence of p53 mutations), but only in a subpopulation of individuals. Thus, the exact role of *H. pylori* in the sequence of events leading to gastric cancer still remains to be elucidated.
